# Supplementary material for: Investigating Bioaccessibility of Advanced Glycation Product Precursors in Gluten-Free Foods Using In Vitro Gastrointestinal System
Source: Medicina (Kaunas). 2023 Aug 30;59(9):1578. doi: 10.3390/medicina59091578 (PMC10535651; doi:10.3390/medicina59091578)
Supplement: Supplementary file 1 [file medicina-59-01578-s001.zip › medicina-2517551-supplementary.pdf]

Supplemantary Table S1: The label information of samples

A. BREAD GROUP

The code of samples that analyzed in HPLC	Contents in the label information of breads
1	Corn starch, water, sugar, vegetable margarine, yeast, thickener (HPMC-Hydroxypromil Methylcellulose), Xanthan gum (E 415), salt, leavening agents (Sodium bicarbonate E450-E 500), preservative (Calcium propionate E 282), antioxidant (ascorbic acid E300)
2	Gluten-free wheat starch, corn starch, pre-sour baker's yeast, (water, buckwheat flour, rice flour, chickpea flour, ground flaxseed meal), potato starch, water, sugar, hazelnut oil, corn flour, salt, egg white powder, consistency enhancers (guar gum, carob gum, xanthan gum, hydroxy propyl methyl cellulose), preservatives (calcium propionate), leavening agents (sodium bicarbonate, sodium acid pyrophosphate).
3	Water, corn starch, rice flour, tapioca starch, potato starch, soy flour, vegetable oil (sunflower), sugar, salt, yeast, thickeners (hydroxypropyl methyl cellulose, carboxy methyl cellulose), preservative (calcium propionate), emulsifier (mono) and diacetyl tartaric acid esters of diglycerides), antioxidant (ascorbic acid).
4	Water, corn starch, rice flour, vegetable fiber (2.3%), sunflower oil, yeast, sugar, thickener (hydroxypropyl methyl cellulose, carboxy methyl cellulose, xanthan gum), salt, preservative (calcium propionate, sorbic acid), acidity regulator (citric acid). May contain traces of nuts, peanuts, eggs, sesame, soy and their products.
5,6	Content information could not be reached.
7	Water, gluten-free flour mixture (corn starch, rice flour), sunflower oil, yeast, salt, thickener (hydroxypropyl methyl cellulose, carboxy methyl cellulose, pectin, xanthan gum), sugar, leavening agents (sodium bicarbonate, sodium acid pyrophosphate), preservative (calcium propionate, sorbic acid), acidity regulator (citric acid), antioxidant (ascorbic acid), vitamins (B1, B2, B6, Niacin), Minerals (Iron, Zinc, Calcium). May contain traces of nuts, peanuts, eggs, sesame, soy and their products.
8	Water, corn starch, sourdough 16% (rice flour, water), rice flour, vegetable fiber (psyllium), 2% flaxseed, 2% soybean flakes, thickener: hydroxypropyl methyl cellulose; sunflower oil, soy protein, yeast, millet flakes 1%, salt, sorghum flour 1%, sugar, citrus fiber, caramelized sugar. May contain traces of lupine. May contain traces of lupine
9	Gluten-free wheat starch, water, sunflower oil, sugar, yeast, salt, flaxseed (2.5%), sesame (2.5%), chia seeds (2.5%), sunflower seeds (2.5%), consistency enhancer (hydroxypropyl methylcellulose,xanthan gum), colorant (caramel), apple fiber, bamboo fiber, psyllium fiber, vitamins and minerals: B1, B2, B6, folic acid, calcium carbonate, ferric pyrophosphate, preservative (calcium propionate), natural flavor transmitters (cumin, coriander)
10	Gluten-free wheat starch, water, sunflower oil, sugar, yeast, salt, thickener (hydroxypropyl methylcellulose, xanthan gum), beet fiber, psyllium fiber, sesame, black cumin, vitamins and minerals: B1, B2, B6, folic acid, calcium carbonate , ferric pyrophosphate, preservative (calcium propionate).
**Total similar ingredients (number of products)**	**Cornstarch (6); rice flour (5), gluten-free wheat starch (3)<br>sunflower oil (6), vegetable margarine (1), hazelnut oil (1)**
**Control groups**	
1,2,3	Bread flour, water, baker's yeast, gluten, whey powder, salt, grape vinegar, food enzymes (fungal alpha amylase, hemicellulase); sugar, ascorbic acid (E300)
4	Bread wheat flour, 20% wheat bran, water, baker's yeast, gluten, whey powder, salt, grape vinegar, food enzymes (fungal alpha amylase, hemicellulase).
5	Wheat flour, drinking water, vegetable oil (palm), stabilizer (glycerol),<br>salt, sugar, acidity regulator (malic acid), leavening agent (disodium dihydrogen diphosphate, sodium hydrogen carbonate), thickener (guar gum), emulsifier (mono and diglycerides of fatty acids, sodium stearol -2-lactylate), yeast extract, preservative (calcium propionate, potassium sorbate)

6	Wheat flour, drinking water, sugar, yeast, vegetable oil (sunflower, canola), gluten, salt, emulsifier (sodium stearol-2-lactylate), preservative (calcium propionate, sorbic acid), antioxidant (ascorbic acid)
7	Wheat flour (gluten), drinking water, yeast, sugar, vegetable oil (indist), vinegar, salt, preservative (calcium propionate, sorbic acid), traces of sesame and milk and dairy products.
8	Whole wheat flour (60%), wheat flour (gluten), drinking water, sunflower seed kernels, yeast, einkorn wheat flour (7%), oatmeal (5%), flaxseed, sesame, corn semolina (5%), herbal oil, bran, rye flour (5%), vinegar, salt, dietary fiber, roasted malt flour (barley), rye pulp, cumin, vitamins (B1, B2, B3, B6, folic acid, B12), minerals (calcium)), zinc, iron)
9	Wheat flour (gluten), drinking water, bran, yeast, dietary fiber (1.1%), salt, preservatives (calcium propionate, sorbic acid), vitamins (B1, B2, B3, B6, folic acid, B12), minerals (calcium, zinc, iron)
**Total ingredients (number of products)**	**Wheat flour (all products), sunflower (3), palm (1) and canola oil (1)**

B) BISCUIT GROUP

The code of samples that analyzed in HPLC	Contents in the label information of biscuits
11	Corn starch, corn flour, sugar, butter, modified tapioca starch, egg, powdered milk, beet syrup, emulsifier: E-472, baking agent: ammonium hydrogen and sodium hydrogen carbonate, salt, natural sweeteners, acid stabilizer: Citric acid.
12	Cocoa cream 39% (sugar, dextrose, lactose (from milk), coconut oil, cocoa powder 12%, palm oil, whole milk powder, natural flavoring: Cocoa, emulsifier: Soy lecithin), corn flour, corn starch, sugar , palm oil, Eggs, whole milk powder, butter, glucose syrup, modified corn starch, rice starch, sea salt, leavening agents: Ammonium hydrogen carbonate, sodium hydrogen carbonate.
13	Rice flour, sugar, vegetable oil (palm, sunflower, cotton, canola), starch (corn), powdered milk, skimmed pasteurized milk 3%, pasteurized egg 3%, minerals (calcium, iron), salt, leavening agent (ammonium hydrogen carbonate), vitamin mixture (b1, b2, b6), flavorings (vanillin, vanilla extract).
14	Corn starch, non-hydrogenated vegetable oil (palm oil), potato starch, invert sugar, salt (2.3%), stabilizer: carboxy methyl cellulose, emulsifier: soy lecithin, leavening agent: sodium hydrogen carbonate, yeast.
15	Milk cream 33% (coconut oil, palm oil, dextrose, lactose (milk), whole milk powder 19%, sugar, emulsifier: soy lecithin, natural flavoring: vanilla), corn starch, sugar, palm oil, corn flour, soy flour, nonfat cocoa powder 3.8%, whole milk powder, potato starch, salt, leavening agents: ammonium hydrogen carbonate and sodium hydrogen carbonate.
**Total similar ingredients (number of products)**	Carbohydrate source: maize flour, maize starch (5) and rice flour and starch (2), potato starch (1), soy flour (1).Sugar source: invert sugar, sugar, or dextrose (5) Oil: Palm oil (3); Coconut oil (2)<br>Powdered milk (4)
**Control group**	
11	Wheat flour, sugar, vegetable oil (palm), invert sugar syrup, whole milk pasteurized (2.5%), leavening agents (ammonium hydrogen carbonate, sodium hydrogen carbonate, disodium diphosphate), whey powder (milk), salt, flavoring donor, flour processing agent (sodium metabisulfite).
12	Wheat flour, sugar, vegetable oils (palm), hazelnut puree (6.6%), invert sugar syrup, starch, leavening agents (ammonium hydrogen carbonate, sodium hydrogen carbonate, disodium diphosphate), whey powder (milk), reduced fat cocoa powder (1.2%), salt, emulsifier (soy lecithin), flavorings, acidity regulator (citric acid), egg, flour processing agent (sodium metabisulfite).
13	Wheat flour, vegetable oil (palm), leavening agents (ammonium hydrogen carbonate, sodium hydrogen carbonate, disodium diphosphate), whey powder (milk), salt, invert sugar syrup, sugar, flavorings (milk), skimmed milk powder, cheese powder (0.2%), flour treatment agent (sodium metabisulfite)
14	Wheat flour (contains gluten), sugar, vegetable oil, reduced palm oil cocoa powder (3.5%), starch, glucose-fructose syrup, leavening agents (sodium carbonate, ammonium hydrogen carbonate), salt, emulsifier (soy lecithin), flavoring . May contain milk.
15	Content information could not be found.
**Total similar ingredients (number of products)**	Carbohydrate source: Wheat flour (5)<br>Sugar source: Invert sugar syrup (5)<br>Oil: Palm oil (5)<br>Powdered milk (5)

C. COOKIE GROUP

The code of samples that analyzed in HPLC	Contents in the label information of cookies
16	Rice flour, sugar, vegetable oil (palm, soybean, sunflower, cotton), whey powder (dairy product), glucose-fructose syrup, egg, starch (corn, potato), flavorings, salt, leavening agent (ammonium hydrogen carbonate), emulsifier (sunflower lecithin).
17	Potato starch, gluten-free wheat starch, egg, sugar, corn starch, hazelnut oil, hazelnut kernels, butter, dark chocolate chips, egg white powder, leavening agent (sodium bicarbonate), thickener (xanthan gum).
18	Rice flour, sugar, vegetable oil (palm, sunflower, cotton, canola), cocoa 4%, whey powder (dairy product), glucose-fructose syrup, pasteurized egg, starch (corn, potato), flavorings, salt, leavening agent (ammonium hydrogen carbonate)
19	Gluten-free flour mixture (corn starch, rice flour), sugar, vegetable oil (varying amounts of palm, shea, cotton, coconut, canola, sunflower, flaxseed, palm, cocoon), egg, chocolate chips (4.6%), sugar, vegetable oil (palm), cocoa powder, emulsifier (soy lecithin), flavor enhancer (vanillin), soluble coffee (1%), emulsifier (sunflower lecithin), flavor enhancers (vanilla, almond), sunflower oil, antioxidant (ascorbic acid). May contain traces of hazelnuts, peanuts, sesame, milk and dairy products.
20	Gluten-free flour mixture (corn starch, rice flour, rice starch), vegetable oil (varying amounts of palm, cotton, sunflower, shea, linseed), sugar, egg, hazelnut (4.6%), emulsifier (sunflower lecithin), sunflower oil, flavor enhancers (vanilla, hazelnut).
21	Gluten-free flour (rice flour, corn starch, sugar, leavening agents (sodium bicarbonate, sodium acid, pyrophosphate), thickeners (pectin, xanthan gum), egg substitute, baking powder, chocolate chips, cocoa, vegetable oil (sunflower oil).
22	Corn starch, vegetable oil (canola, sunflower), sugar, low protein chocolate (5%), leavening agent (sodium bicarbonate, sodium diphosphate, sodium acid pyrophosphate), thickener (xanthan gum), acidity regulator (glycerol monostearate, glucano delta- lactone), preservative (calcium propionate), vitamins and minerals: B1, B2, B6, folic acid.
23	Powdered sugar [sugar, corn starch (max. 3%), rice flour, potato starch, vegetable oil [palm, sunflower, rapeseed, emulsifier (mono and diglycerides of fatty acids)], coconut (11%), drinking water, consistency enhancer (xanthan gum), leavening agents (sodium acid pyrophosphate, sodium hydrogen carbonate), sea salt, emulsifier (soy lecithin), flavoring (coconut).
24	Corn starch, vegetable oil (canola, sunflower), sugar, raisins (5%), leavening agent (sodium bicarbonate, sodium diphosphate, sodium acid pyrophosphate), thickener (xanthan gum), acidity regulator (glycerol monostearate, glucano delta-lactone)), preservative (calcium propionate), vitamins and minerals: B1, B2, B6, folic acid.
25	Chickpea powder, white chocolate, powdered sugar, vegetable oil.
26	* Content information could not be found.
**Total similar ingredients (number of products)**	Rice flour and cornstarch (8),<br>Palm, sunflower, cotton, canola oil (6)<br>Invert sugar/glucose fructose syrup (4)
**Control group**	
16,17	Wheat flour (gluten), vegetable oil (palm), granulated sugar, starch (corn), butter (10%), egg, powder risers (sodium acid pyrophosphate, sodium bicarbonate), vanilla, (butter flavoring)
18	Wheat flour, vegetable oils (pal, palm kernel, fully hydrogenated palm, fully hydrogenated palm kernel), sugar, invert sugar syrup, eggs, applesauce, hazelnuts, reduced-fat cocoa powder, salt, leavening agents (ammonium hydrogen carbonate, sodium hydrogen carbonate) , disodium diphosphate), starch, skimmed milk powder, whey powder (milk), flavorings, whole milk powder, emulsifiers (soy lecithin, polyglycerol polyricinoleate).
19	* Content information could not be found.
20	Wheat flour, sugar, vegetable oil (palm), hazelnut (4.8%), leavening agents (ammonium hydrogen carbonate, disodium diphosphate, sodium hydrogen carbonate), invert sugar syrup, salt, whole milk powder, flavorings, egg, emulsifier (soy lecithin)
21,22	Wheat flour, vegetable margarine (date, soy, canola, cotton, sunflower oil, potassium sorbate (E202), citric acid, beta carotene, butter flavoring), grape (11%) sorbic acid (from raw material), sugar, egg, vegetable oil (sunflower; antioxidant, anti-foaming), powdered sugar, vanilla, baking powder (raising (E450), (E500), anti-caking (E170), sulphite).
23-24	Content information could not be found.
**Total similar ingredients (number of products)**	**Wheat flour (whole), palm oil, (3) Sunflower oil (2), butter (2),**<br>**Invert sugar (2)**

Supplemantary Table S2: The energy and nutrient label information of samples

A. BREAD GROUP

The energy and nutrient label information of breads							
**Gluten-free**	**Carbohydrate (g)**	**Sugar (g)**	**Fat (g)**	**Saturated fat (g)**	**Protein (g)**	**Fiber (g)**	**Salt (g)**
1	49,4	3,3	4,2	2,1	0,5	0,1	1,4
2	54,3	2,3	1,23	0,6	1,7	3,2	0,7
3	49,0	1,6	1,2	0,1	4	*	1
4	52,1	1,3	3,5	0,3	0,9	2,5	1,4
5	49,4	3,30	4,2	2,1	0,5	0,1	1,4
6	47.8	0,5	1,8	0,2	0,7	*	1,1
7	54,5	2,9	2,0	1,3	1,6	*	0,9
8	44	6,3	2,4	0,4	3,8	6,7	1,1
9	47,8	2,2	10,4	0,41	7,6	2,88	1,7
10**	55,8	2,2	4,9	0,41	0,2	4,8	1,7
**Control**							
1,2,3	49,4	2,9	3,2	0,2	8,2	4,6	0,5
4	43,3	2,8	1,4	0,3	9,8	6,8	0,7
5	47,7	3,3	6,2	3,2	8,9	3,4	0,9
6	57,2	3,0	2,5	1,9	7,1	2,3	0,9
7	54,5	2,9	2,0	1,3	6,8	2,3	0,9
8	37,9	2,6	1,4	0,8	11,9	6,6	0,9
9	44,5	3,5	1,5	0,4	6,5	7,5	0,9

Control: gluten-containing. Content information could not be reached.**Product number 10 does not have a control group

B) BISCUIT GROUP

The energy and nutrient label information of biscuits							
**Gluten-free**	**Carbohydrate (g)**	**Sugar (g)**	**Fat (g)**	**Saturated fat (g)**	**Protein (g)**	**Fiber(g)**	**Salt (g)**
11	80,0	16,0	14,0	9,4	2,7	0,5	1,0
12	71	34	21	13	3,9	1,9	0,5
13	75,2	20,6	14,1	7,3	5,8	1,0	0,4
14	72	1	21	9,9	0,9	0,7	2,3
15	66	33	24	14	5,8	2,5	0,8
**Control**							
11	72	21	14	6,8	8,1	1,8	0,7
12	62,0	26,0	26,0	11,0	5,6	2,5	0,7
13	71,5	24,3	14,5	7,1	5,3	2,6	1,0
14	61,0	7,3	20,0	9,6	7,7	1,8	3,0
15	68,0	37,5	21	10,5	4,9	2,5	1,1

Control: gluten-containing

B) COOKIE GROUP

The energy and nutrient label information of cookies							
**Gluten-free**	**Carbohydrate (g)**	**Sugar (g)**	**Fat (g)**	**Saturated fat (g)**	**Protein (g)**	**Fiber(g)**	**Salt (g)**
16	65,8	21,1	29,5	16,5	0,3	0,2	0,3
17	68,4	19,3	22,2	7,8	4,5	1,8	0
18	69,3	27	20,2	10,6	4,5	1,2	0,7
19	58,7	17,5	24,9	13,1	2,0	*	0
20	62,3	12,3	32,3	15,4	2,3	*	0
21	72	*	19	*	4	*	0,1
22	57,2	12,5	27,1	0,5	0,5	7,2	0,1
23	61,4	26,8	23,5	15,6	3,1	7,0	0,4
24	61,4	12,5	27,1	14,9	0,3	5,2	0,1
25	*	*	*	*	*	*	*
26	58,4	*	16	3	2,1	0,3	1,8
**Control**							
16,17	42,3	18,0	25,0	13,8	2,3	*	0,9
18	65,0	24,0	23,0	11,0	6,6	1,7	0,7
19	50,3	17,1	14,9	12,2	5,5	*	0,1
20	65,0	24,0	21,0	9,0	7,1	1,9	0,9
21,22	66	30	22	12	5,7	1,4	0,83
23	15,3	5,3	7,3	4,0	1,6	0,3	0,1
24	66,6	24,3	21,6	7,5	7,9	*	1,3

Control: gluten-containing *Content information could not be reached . Product number 25 and 26 does not have a control group
